# Colorimetric Determination of Hypochlorite Based on the Oxidative Leaching of Gold Nanorods

**DOI:** 10.3390/ma11091629

**Published:** 2018-09-06

**Authors:** Yurong Ma, Yingyi Zhu, Benzhi Liu, Guixiang Quan, Liqiang Cui

**Affiliations:** 1 School of Environmental Science and Engineering, Yancheng Istitute of technology, Yancheng 224051, China; yingyi.zhu@outlook.com (Y.Z.); benzhiliu@163.com (B.L.); qgx@ycit.cn (G.Q.); lqcui8@gmail.com (L.C.); 2 Key Laboratory for Advanced Technology in Environmental Protection of Jiangsu Province, Yancheng Institute of Technology, Yancheng 224051, China

**Keywords:** gold nanorod, colorimetric determination, hypochlorite

## Abstract

Hypochlorite plays a critical role in killing microorganisms in the water. However, it can also cause cardiovascular diseases, neuron degeneration, and cancer to humans. Although traditional methods feature excellent sensitivity and reliability in detecting hypochlorite, the expensive instruments and strict determination conditions have limited their application in environmental analysis to some extent. Thus, it is necessary and urgent to propose a cheap, facile, and quick analytical assay for hypochlorite. This paper proposes a colorimetric assay for hypochlorite utilizing gold nanorods (AuNRs) as the nanoreactor and color reader. The AuNRs were acquired via a reported seed-mediated method. NaClO with strong oxidation property can cause the etching of gold from the longitudinal tips of AuNRs, which could shorten the aspect ratio of AuNRs, decrease the absorption in the UV–Vis spectrum and also induce the solution color changing from red to pale yellow. Thus, according to the solution color change and the absorbance of longitudinal surface plasmon resonance of AuNRs, we established the calibration curve of NaClO within 0.08 μM to 125 μM (∆Abs = 0.0547 + 0.004 C_NaClO_, R^2^ = 0.9631). Compared to traditional method, we obtained the conversion formula between the concentration of residual-chlorine in tap water and the concentration of hypochlorite detected by the proposed colorimetric assay, which is C_residual-chlorine_ = 0.24 C_NaClO_. Finally, the real application of the colorimetric assay in tap water was successfully performed, and the accuracy of the colorimetric method can reach from −6.78% to +8.53%.

## 1. Introduction

In the human immune system, hypochlorite is famous for its special antibacterial properties [1,2]. Chlorination disinfection has been added legally and illegally in water treatment, household bleach, and beverage industries [3,4,5]. However, the excess residual chlorine in organism can induce cardiovascular diseases, neuron degeneration and cancer [6,7,8]. According to previous report, hypochlorite (HClO) and sodium hypochlorite (NaClO) have already been presented in drinking water, beverages, infant formula and raw meat in the range of 10^−5^ M to 10^−2^ M, which largely threats the health of humans [7]. Enormous efforts have been made to explore the measurement of HClO and NaClO, including gas chromatography-flame ionization detection (GC-FID), fluorescence technique based on gold nanoclusters or organic probe, multi-syringe flow injection analysis system, and electrochemical methods [7,9,10,11,12,13,14]. However, most of the present techniques usually demand expensive instruments, harsh analytical conditions, and prolonged reaction time. Therefore, it is emergent to establish an easy, inexpensive, and quick analytical methods for hypochlorite in water quality and food safety. 

Metallic nanoparticles, such as gold nanoparticles (AuNPs) and silver nanoparticles (AgNPs), have acquired great concern due to their unique distance-, size- and shape-dependent physical properties, and show enormous applications in the field of colorimetric assay. To date, AuNPs- and AgNPs-based colorimetric sensors have already realized the determination of DNA [15], pesticide [16], methionine [17], glucose [18], metal ions [19,20], tyrosinase [21], disease biomarkers [22], thiourea [23], and small molecules [24,25]. The existing colorimetric method mainly rely on the distance-dependent color change of the metallic suspensions, and require the further modification of nanoparticles, which would prolong and hinder the whole detection process. Different from AuNPs and AgNPs, gold nanorods (AuNRs) possess two typical absorption bands, one is the transverse surface plasmon resonance (TSPR) absorption around 510 nm, the other is the longitudinal surface plasmon resonance (LSPR) absorption [26], and the LSPR absorption of AuNRs have higher extinction coefficients than AuNPs and AgNPs, which is more favourable as colorimetric sensors. Furthermore, the LSPR absorption of AuNRs mainly depend on the aspect ratio of AuNRs and can be varied from 600 to 900 nm by oxidative reaction [27]. The longitudinal phase of AuNRs have high reaction activities, thus the longitudinal tips of AuNRs can be oxidized from Au(0) to Au(I) at certain conditions, and the leaching of gold would induce the shortening aspect ratio of AuNRs, blue-shift of the LSPR absorption as well as obvious color change of the AuNRs suspension, which is sufficient for colorimetric method. Actually, there have been colorimetric sensors for metal ions [27,28], I^−^ [29], acetylcholinesterase [30], and small molecules [31,32,33,34], based on the oxidative etching of gold nanoparticles. Such colorimetric sensors do not demand the complex modification of gold nanoparticles, which supply a simple and time-saving platform for signalling target compound. 

Little achievements have been monitored toward the direct determination of small molecular in environmental samples, owing to their low content, complex matrix compose, and various interfering ions. However, based on the selective reaction between oxidative pollutant and gold element, we developed the possibility for the tracking of NaClO utilizing unlabelled AuNRs as colorimetric reader. We synthesized the AuNRs via the traditional seed-mediated method. NaClO, with strong oxidation property and extensively utilization in commercial disinfectant, can cause the etching of gold element from the tips of AuNRs, decrease the aspect ratio of AuNRs, and also induce the solution colour changing from red to pale yellow. Furthermore, common interfering substance do not disturb the detection of NaClO. Thus, combing the solution colour change and the LSPR absorption of AuNRs suspension, we established a simple and sensitive colorimetric assay for NaClO. In comparison with traditional method, we got the conversion formula between the concentration of NaClO and the amount of residual chlorine in tap water. Finally, based on the simple and sensitive colorimetric method, we achieved the detection of residual chlorine in fresh tap water, which showed great potential promise in environmental application field.

## 2. Materials and Methods

### 2.1. Materials

Standard reagent sodium hypochlorite (NaClO, 50 mL 0.10 M) was purchased from Beijing Putian Genesis Biotechnology Co., Ltd. (Beijing, China) Tetrachloroauricacid hydrate (HAuCl_4_·4H_2_O), ascorbic acid (AA), sodium borohydride (NaBH_4_), silver nitrate (AgNO_3_), hexadecyltrimethylammoniumbromide (CTAB), ferric chloride hexahydrate, calcium chloride, magnesium sulfate, copper sulphate, aluminum nitrate, zinc nitrate, cobalt chloride, hydrochloric acid, nitric acid were purchased from Sinopharm Chemical Reagent Co. Ltd. (Shanghai, China). 

All reagents were analytical grade and without further purification. Ultrapure water was prepared with a Milli-Q water purification system (Millipore, Bedford, MA, USA).

### 2.2. Apparatus and Instruments

UV–Vis spectra were monitored by nucleic acid/protein analyzer DU 800 (Beckman Instruments, Inc., Brea, CA, USA). The optical images were obtained by Canon EOS 700D digital camera (Oita Prefecture, Kyushu, Japan). Transmission electron microscope (TEM) measurements were measured by a JEOL JEM-2100F (Akishima, Tokyo, Japan). X-ray Diffraction (XRD) tests were operated with the X’Pert3 Powder (PANalytical B.V., Almelo, Holland).

### 2.3. Synthesis of AuNRs

In this paper, AuNRs were synthesized via a reported seed-mediated growth method [26,35,36]. The concentration of original AuNRs (0.9 nM) was calculated by Lambert-Beer law based on the extinction coefficient of 4.6 × 10^9^ M^−1^cm^−1^ [37].

### 2.4. Optimization of Experimental Conditions

#### 2.4.1. Concentration of AuNRs

The original of 0.9 nM AuNRs were diluted with different volume of ultrapure water to acquire 0.4 nM, 0.3 nM, and 0.2 nM AuNRs correspondingly. Then 1.0 mL of the diluted AuNRs solution, 40 μL of 0.1 M HCl, 30 μL of 1 mM NaClO were added into in a 1.5 mL centrifugal tube in sequence. 

#### 2.4.2. Incubation Time 

1.0 mL of 0.4 nM AuNRs, 50 μL of 0.1 M HCl, and 50 μL of 1 mM NaClO were added in a 1.5 mL centrifugal tube. And the UV–Vis spectrum of the mixture was measured different incubation time, including 0, 5, 10, 20, 30, 45, 60 and 120 min.

#### 2.4.3. Selectivity of the Colorimetric Sensor

One millilitre of 0.4 nM AuNRs, 50 μL of 0.1 M HCl, and the corresponding interfering substance were added in a 1.5 mL centrifugal tube. Photographs and the UV–Vis spectrum of the solution were all recorded after 5 min.

#### 2.4.4. Colorimetric Detection of NaClO in Real Tap Water

One millilitre of 0.4 nM AuNRs, 50 μL of 0.1 M HCl, and different volume of 1 mM NaClO were added into the centrifugal tube and the overall amount of NaClO in the tube were settled to be 0, 0.08, 0.42, 0.83, 4.17, 8.33, 16.7, 25.0, 41.7, 66.7, 75, 83.3, 108 and 125 μM correspondingly. Photographs and the UV–Vis spectra of the solutions were all measured after 5 min and each concentration was measured two parallel groups.

Four-hundred forty-four microlitres of 0.9 nM AuNRs, 120 μL of fresh collected tap water, 456 μL of ultra-pure water and 50 μL of 0.1 M HCl were mixed in a 1.5 mL centrifugal tube, and the UV–Vis spectrum of the reaction system was recorded after 5 min. The tap water was obtained from our own laboratory (sample 1) and residential areas (sample 2). 

## 3. Results and Discussion

### 3.1. Mechanism and Characterization of the Colorimetric Assay

**Figure 1:**
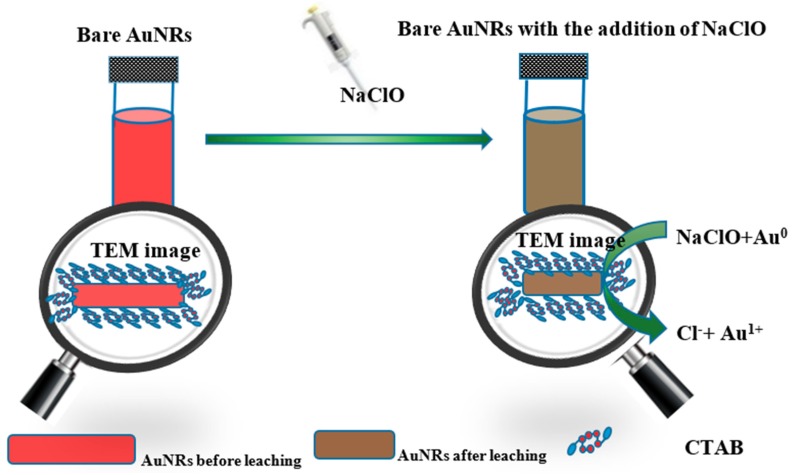
Mechanism of colorimetric determination of NaClO based on the oxidative leaching of AuNRs.

**Figure 2:**
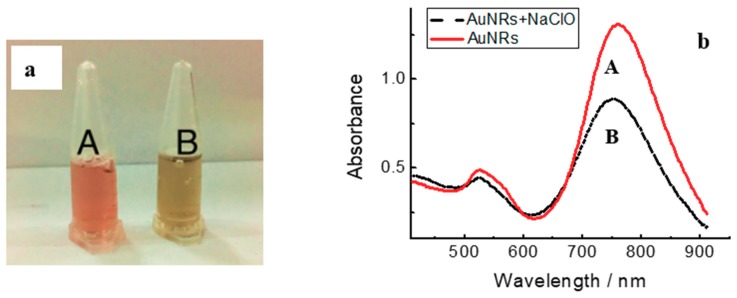
Photographs (**a**) and UV–Vis spectrum (**b**) of bare AuNRs (A) and with the addition of NaClO (B).

The standard electron potentials of Au(I)/Au(0) is 1.691 eV. However, in the presence of Br^−^ of CTAB, the standard electron potentials of Au(I)/Au(0) can reduced from 1.691 to 0.959 eV. The standard electron potentials of HClO + H^+^ + 2e = Cl^−1^ + H_2_O is 1.49 eV, which is higher than Au(I)/Au(0) in the presence of Br^−^ and provides the possibility of HClO oxidizing gold element [27,28]. As the oxidative reaction undergoes (Figure 1), the chemical etching prefers to occur along the longitudinal direction of AuNRs, which can be explained by less surface passivation or higher reactivity at the tips of the AuNRs. For the sake of studying the feasibility of the proposed strategy, AuNRs were firstly synthesized with the traditional seed-mediated method. As shown in Figure 2a, the bare AuNRs exhibit a red colour, which is easy to recognize and favourable for colorimetric reader. While NaClO was coexisted with the bare AuNRs, the suspension colour changed from red to pale yellow, which suggested the possible etching reaction between AuNRs and NaClO. As shown in the UV–Vis spectrum, the bare AuNRs show two typical absorption peaks at 514 nm and 760 m in Figure 2b, corresponding to TSPR absorption and LSPR absorption of AuNRs [26], which suggested the successful synthesis of AuNRs. After the addition of 80 μM NaClO, the LSPR wavelength of AuNRs changed from 760 to 735 nm, and the LSPR absorbance decreased from 1.321 to 0.889. The LSPR absorption of AuNRs highly rely on the aspect ratio of AuNRs, thus the blue-shift and the decreased absorbance of LSPR absorption both suggested the shortening aspect ratio of AuNRs. The TSPR absorption also showed a slight change, which is also caused by the decreased aspect ratio of AuNRs [27].

**Figure 3:**
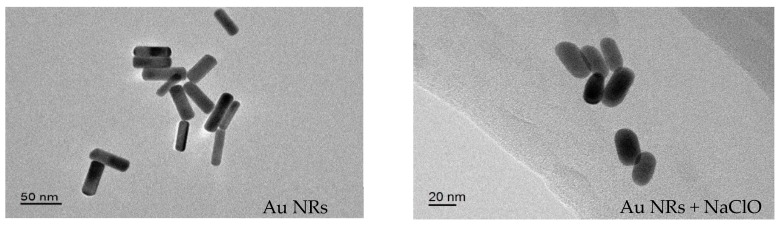
TEM image of bare AuNRs (**left**) and with the addition of NaClO (**right**).

TEM measurement were further studied as shown in Figure 3. The synthesized AuNRs show an average longitudinal length of 48 ± 2 nm and a transverse diameter of 14 ± 2 nm. While after the addition of 80 μM NaClO, the longitudinal length of AuNRs have decreased to 28 ± 1 nm and the transverse diameter still kept 14 ± 2 nm. These phenomena further proved the proposed mechanism that the oxidative leaching reaction between AuNRs and NaClO was expected to occur along the longitudinal tips of AuNRs, which might because the longitudinal phase of AuNRs have high reaction activities. The aspect ratio (length/diameter) of AuNRs are reduced from 3.43 to 2.00 due to the etching reaction, which explained the distinct blue-shift and decreasing absorbance of LSPR in the UV–Vis spectrum. Thus, according to the change of LSPR absorption and the solution colour, we could bring forward a colorimetric sensor for NaClO. 

**Figure 4:**
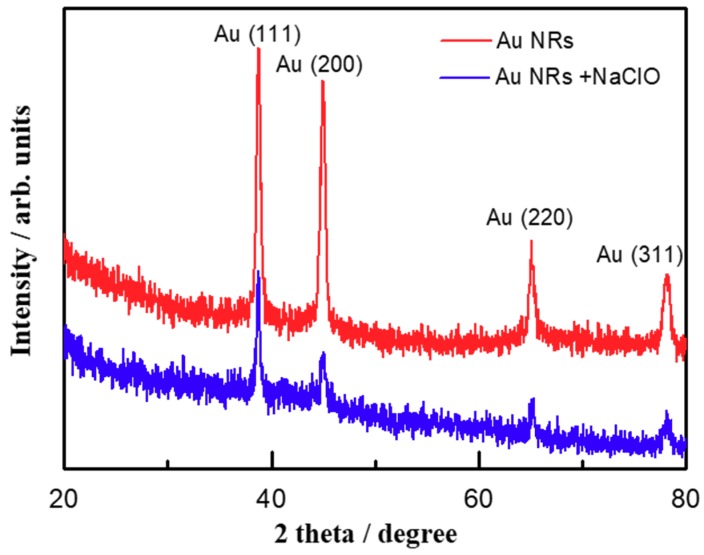
XRD spectrum of bare AuNRs (red) and with the addition of NaClO (blue).

X-ray diffraction (XRD) was also carried out to investigate the structural change related with the oxidative etching reaction. As shown in Figure 4, the initial AuNRs exhibited four strong and sharp diffraction lines at 38.45°, 44.73°, 64.68° and 77.87°, respectively, correspond to (111), (200), (220), (311) reflections of the face-cantered cubic structure of metallic Au (JCPDF No. 04-0784) [38]. Compared with the initial AuNRs, the typical peaks of metallic gold still exist in the XRD spectrum after 30 min reaction with NaClO, but with dramatically decreased intensity, which means the original AuNRs have larger size and again proved the etching reaction between AuNRs and NaClO [39].

### 3.2. Optimization of Experimental Conditions

In order to establish a convenient and sensitive colorimetric method for NaClO, reaction conditions including concentrations of AuNRs, concentrations of HCl, and incubation time were investigated in details with the UV–Vis spectrum. 

**Figure 5:**
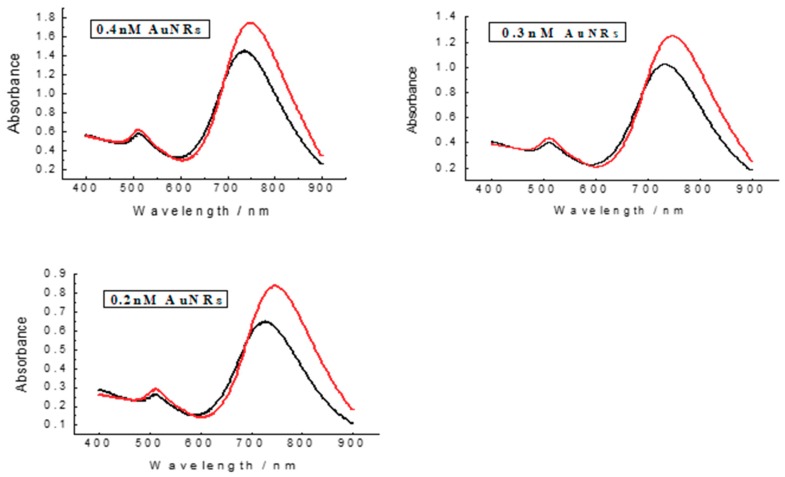
UV–Vis spectrum of different concentrations of bare AuNRs (red) and with the addition of 30 μM NaClO (black). Other experimental conditions, HCl (4 mM), incubation time (5 min).

Different from spherical gold nanoparticles, the extinction coefficient for LSPR band of AuNRs is highly rely on the aspect ratio of AuNRs, and the extinction coefficient of prepared AuNRs is assigned to be 4.6 × 10^9^ M^−1^ cm^−1^ according to previous report [37], and the concentration of original AuNRs is calculated to be 0.9 nM by Lambert-Beer law. As the colorimetric reader, the amount of AuNRs would directly affect the signal response of the sensor toward NaClO, thus the concentration of AuNRs were studied in Figure 5. The initial AuNRs of 0.9 nM were diluted by ultrapure water to acquire different concentrations of AuNRs of 0.4 nM, 0.3 nM, and 0.2 nM correspondingly. The UV–Vis spectrum was monitored and the absorbance change of AuNRs LSPR absorption before and after the addition of 30 μM NaClO were listed in Table 1 (∆Abs = A_before_ − A_after_). In accordance with the prior results, all three LSPR absorption of AuNRs decreased with the addition of NaClO, and the optimal AuNRs concentration was settled to be 0.4 nM, at which the colorimetric sensor exhibited the strongest signal response to 30 μM NaClO. Furthermore, compared to 0.2 nM and 0.3 nM of AuNRs, 0.4 nM of AuNRs have brighter colour, which is more favourable for the colour identification. 

**Figure 6:**
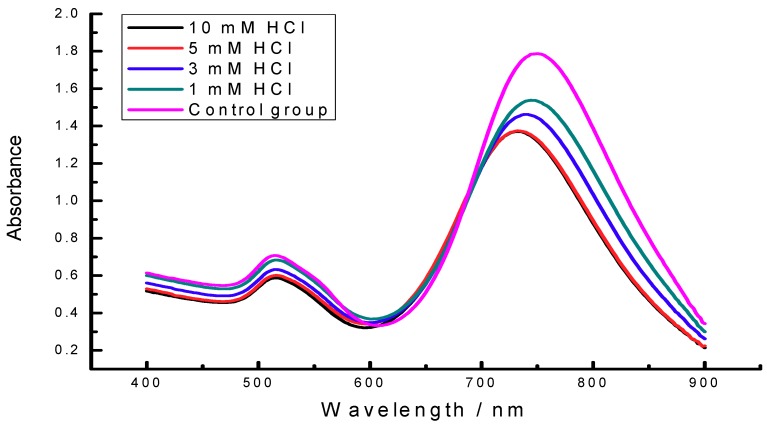
The effect of pH on the detection assay. Experimental conditions, AuNRs (0.4 nM), NaClO (30 μM), incubation time (5 min), and different concentrations of HCl.

**Figure 7:**
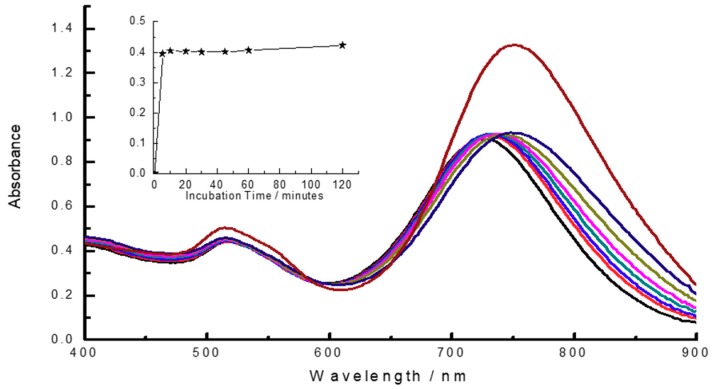
UV–Vis spectrum of the reaction system at different incubation time, from upper to lower: (**1**) 0 min; (**2**) 5 min; (**3**) 10 min; (**4**) 20 min; (**5**) 30 min; (**6**) 45 min; (**7**) 60 min, and (**8**) 120 min. The inset describes the kinetics/time curve at certain time interval in 120 min. Experimental conditions, 0.4 nM AuNRs, 50 μM NaClO, and 5 mM HCl.

The oxidative leaching reaction of gold from AuNRs is inclined to occur at acidic conditions [27], it is assumed that the addition of HCl would accelerate the reaction rate. Therefore, the effect of HCl dosage on the colorimetric sensor is studied by adding different concentrations of HCl in Figure 6 and Table 2. The data suggested that the optimized amount of HCl was 5 mM (pH 2.49), at which the colorimetric sensor displayed the largest LSPR absorption change and the oxidative etching reaction between AuNRs and NaClO could be reacted at a quick reaction rate. The kinetics/time curve were recorded after the addition of 50 μM NaClO into the AuNRs solution at certain time interval in 120 min. As shown in the inset of Figure 7, the colorimetric system almost can achieve the point of reaction balance in 5 min, which was settled to be the incubation time. Under the optimal reaction conditions (0.4 nM AuNRs, 5 mM HCl, incubation time 5 min), the UV–Vis spectrum and photographs were all taken at 5 min, which means the colorimetric detection of NaClO could be accomplished in 5 min. 

### 3.3. Selectivity of the Optical Sensor

**Figure 8:**
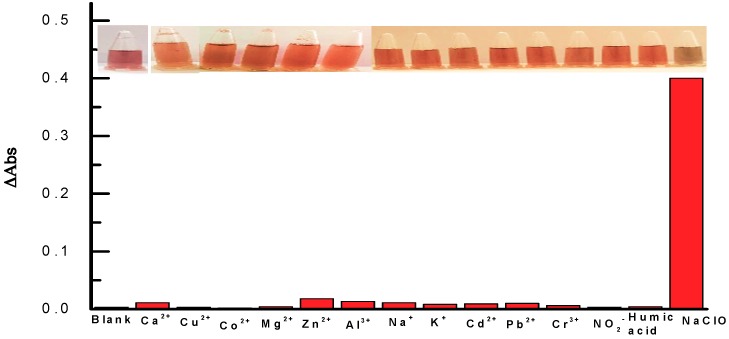
Photographs and absorbance change (ΔA) of the reaction system with the addition of corresponding interfering substances, from left to right:blank, 1 mM of (Ca^2+^, Cu^2+^, Co^2+^, Mg^2+^, Zn^2+^, Al^3+^, Na^+^, K^+^), 1 μM of Cd^2+^, 2 μM of (Pb^2+^, Cr^3+^, NO_2_^−^), 8 μM of humic acid, and 80 μM of NaClO. Experimental conditions: 0.4 nM AuNRs, 5 mM HCl, incubation time: 5 min, different concentrations of interfering substances.

Considering the complex matrix composition of the real water samples, the selectivity of the optical sensor was investigated. Under the optimal conditions, several common interfering substances [20] (Ca^2+^, Cu^2+^, Co^2+^, Mg^2+^, Zn^2+^, Al^3+^, Na^+^, K^+^, Cd^2+^, Pb^2+^, Cr^3+^, NO_2_^−^, humic acid) were added into the reaction system, and the incorporation of them did not induce the colour change and LSPR decreasing of the AuNRs suspension (Figure 8), indicating that none of them would interfere the determination of NaClO with the proposed method. 

### 3.4. Colorimetric Sensing of NaClO 

**Figure 9:**
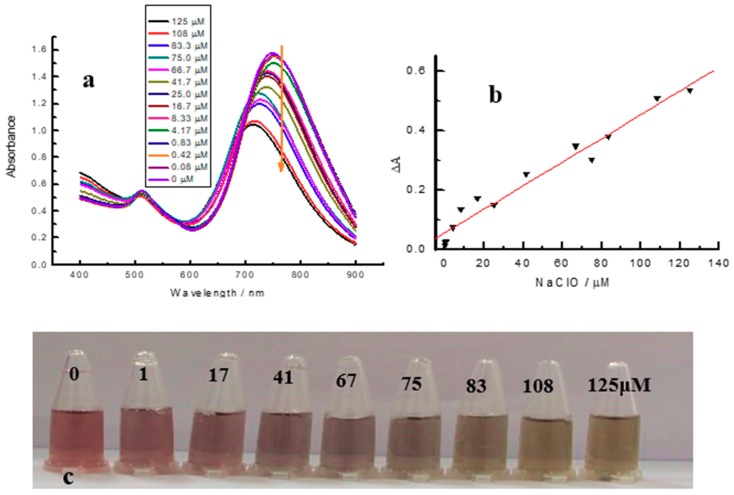
(**a**) UV–Vis spectrum of the reaction system with different concentrations of NaClO; (**b**) the plot of ∆Abs against C_NaClO_ for NaClO analysis; and (**c**) photographs of the reaction system with different concentrations of NaClO. Experimental conditions, 0.4 nM AuNRs, 5 mM HCl, incubation time: 5 min, different concentrations of NaClO as shown in the figure above.

In order to realize the quantitative determination of NaClO in real water samples, the calibration curves of NaClO was explored by adding different amount of NaClO into the AuNRs suspension. As shown in Figure 9c, with the amount of NaClO increased from 1 to 125 μM, the AuNRs solution colour gradually changed from red, light purple to pale yellow, which suggested that we can achieve the semi-detection of NaClO (from 1 to 125 μM) with the naked eye. In the UV–Vis spectrum (Figure 9a), the increasing NaClO resulted in the wavelength blue-shift and the decreased absorbance of the LSPR absorption, which inferred the decreased aspect ratio of AuNRs. Furthermore, ∆Abs is linear with the concentration of NaClO within 0.08 μM to 125 μM, while the calibration curve is ∆Abs = 0.0547 + 0.004 C_NaClO_ (R^2^ = 0.9631). 

### 3.5. Determination of Residual Chlorine in Real Water Samples

In order to investigate the potential application of this colorimetric assay in real water, we compared the proposed colorimetric method with a classical *N*,*N*-Diethy-*p*-phenylenediamine (DPD) Spectrophotometric method. The concentration of free residual chlorine (containing ClO^−^, Cl_2_) in the fresh tap water were first determined according to the Chinese National Standard method (CNS, GB/T 5750. 11-2006), which using DPD as the chelating colorant and the concentration of residual chlorine was finally obtained as 2.28 μM. The colorimetric assay was also utilized to detect the amount of free residual chlorine in the tap water sample. After adding the diluted tap water into AuNRs solution, the suspension colour changed from red to dark brown in 5 min and there was also subtle LSPR change in the UV–Vis spectrum, which may suggest the presence of oxidative chlorine in the fresh tap water. According to the calibration curve in Figure 9b, the amount of NaClO in tap water was calculated to be 9.6 μM, thus the conversion formula of the concentration of residual chlorine between DPD assay and AuNRs assay is C_residual-chlorine_ = C_DPD assay_ = (2.28/9.6) × C_AuNRs assay_ = 0.24 C_NaClO_. Finally, the conversion formula was studied with two tap water samples to validate the reliability of the proposed assay, and the accuracy of the colorimetric method can reach +8.53% and −6.78%, respectively, which demonstrating good method precision (Table 3).

## 4. Conclusions

In summary, we proposed a new colorimetric sensor for hypochlorite based on the oxidative etching reaction between hypochlorite and AuNRs. NaClO, with strong oxidation property, can cause the etching of gold element from the longitudinal tips of AuNRs, decrease the aspect ratio of AuNRs, and also induce the solution to change colour from red to pale yellow, which can be used for the colorimetric analysis of NaClO. This sensor presents the following advantages. First, the determination of NaClO could be fulfilled in 5 min, which is convenient for emergency monitoring. Second, bare AuNRs without further modification can be directly used as a nanoreactor and colour reader, which simplifies the operation. Third, the sensor possesses excellent sensitivity and selectivity. Finally, compared to the traditional method, we have finally accomplished the determination of residual chlorine in real tap water based on the proposed colorimetric assay, which broadens the potential application in real natural water systems.

## Figures and Tables

**Table 1:** ∆Abs of AuNRs in Figure 5 induced by NaClO.

Of AuNRs/nM	∆Abs
0.4	0.299
0.3	0.225
0.2	0.188

**Table 2:** ∆Abs of AuNRs in Figure 6 induced by HCl.

Concentration of HCl/mM	∆Abs
1	0.25
3	0.326
5	0.413
10	0.411

**Table 3:** Determination of free residual chlorine in fresh tap water.

Cresidual-Chlorine/μM-Determined by DPD Assay		Cresidual-Chlorine/μM-Determined by AuNRs Assay	Accuracy of the Colorimetric Method
Sample 1	2.11	2.29	+8.53%
Sample 2	1.92	1.79	−6.78%
